# VectorSage: enhancing PubMed article retrieval with advanced semantic search

**DOI:** 10.1093/bioadv/vbag116

**Published:** 2026-04-24

**Authors:** Yasas Wijesekara, Rahul Brahma, Mehdi Lotfi, Marcus Vollmer, Lars Kaderali

**Affiliations:** Institute of Bioinformatics, University Medicine Greifswald, Greifswald, 17475, Germany; Institute of Bioinformatics, University Medicine Greifswald, Greifswald, 17475, Germany; Institute of Bioinformatics, University Medicine Greifswald, Greifswald, 17475, Germany; Institute of Bioinformatics, University Medicine Greifswald, Greifswald, 17475, Germany; Institute of Bioinformatics, University Medicine Greifswald, Greifswald, 17475, Germany

## Abstract

**Motivation:**

The exponential growth of academic literature has presented unprecedented opportunities. However, it also underscores the need for advanced search methodologies to support efficient knowledge discovery. While effective for structured queries, traditional keyword-based search engines often struggle with the inherent variability of language, where the same concept can be expressed in many ways, leading to incomplete or imprecise retrieval of relevant research. Another issue that must be considered is that of lexical ambiguity, such as polysemy or homonymy, whereby several words and abbreviations can have multiple meanings. This results in items placed in the results list that are irrelevant to the search context. Recent advances in natural language processing have enabled semantic similarity techniques that move beyond basic text matching toward context-aware search.

**Results:**

We developed VectorSage (https://vectorsage.nube.uni-greifswald.de), an advanced biomedical search system for retrieving PubMed abstracts using a hybrid approach that combines term relevance scoring with embedding-based semantic similarity. VectorSage employs a global ranking mechanism to enhance further search relevance by sorting the retrieved documents, ensuring a balance between semantic relevance and keyword specificity. This method enables efficient literature exploration and knowledge discovery.

## 1 Introduction

Over the last century, there has been no sign of a reduction in the growth rate of the publication of scientific literature (Larsen 2010). Concurrently, the emergence and the adoption of new platforms such as conference proceedings, preprint archives, and blogs have significantly increased, further contributing to the already exponential growth in the overall publication of journals and academic literature (Price 2019). This growth presents significant challenges in information retrieval, limiting researchers’ ability to efficiently find relevant prior research in specific and/or across disciplines. As a result, many studies are conducted in isolation from existing evidence bases, leading to repetitive research and misallocation of scientific resources and hindering knowledge integration and the cumulative advancements of scientific knowledge. Failure to integrate empirical or theoretical analyses limits the relevance, reducing the contextual validity and translational potential. This highlights a paradox in academia: while information is abundant, the translation into actionable knowledge remains limited (Branin and Case 1998).

While access to large-scale information resources is empowering, unless we can extract actionable knowledge from it, it is of no benefit, which necessitates an efficient tool for navigating and extracting meaningful insights from this large amount of information. Traditional keyword-based search engines have been effective on smaller datasets; however, on large volumes of data, they become increasingly impractical. They often struggle with the inherent variability of language, where the same concept can be expressed in many ways, leading to incomplete or imprecise retrieval of relevant research. Another issue that must be considered is that of lexical ambiguity, such as polysemy or homonymy, whereby several words and abbreviations can have multiple meanings. As a result, search results contain items that do not pertain to the search context. Moreover, changes in user behaviors are also evolving; their search query patterns have particularly shifted toward more natural language input, and thus, the conventional approach is often unable to capture the meaning and expression of users’ queries, limiting their effectiveness in providing contextually relevant results (Negi and Kumar 2014, Sangeetha *et al.* 2024, Rahman n.d.). For example, PubMed’s fuzzy string-matching algorithm, the Automatic Term Mapping system (ATM) (Thirion *et al.* 2009), a citation sensor, and their Best Match ranking algorithms are designed to optimize search relevance, but often struggle with inconsistencies in term assignments, and have not been able to keep up with changing users’ behaviors (Portaluppi 2007, Massonnaud *et al.* 2020).

Recent work in information retrieval has focused on developing domain-specific search filters to improve sensitivity and specificity in systematic reviews (Salvador-Oliván *et al.* 2021). In parallel, an effort was also made toward extracting the possible semantic meaning of keywords, which outputs a query expression of the keywords into a natural language with intended semantic information, showing better performance than traditional approaches (Trillo *et al.* 2007). Knowledge graph embedding models have also been tested on articles indexed in PubMed, demonstrating potential for semantic-aware search (Ebeid 2022). Furthermore, a comparative evaluation of 28 academic search systems against 27 functionality criteria revealed substantial variation in performance, with approximately 50% of these tested search systems exhibiting critical performance limitations in query formulation and the correct interpretation of the query (Gusenbauer and Haddaway 2020). These findings highlight the limitations of the conventional approach and necessitate a tool that supports the shift towards using search engines with more natural language understanding, allowing users to communicate comfortably instead of strictly following system-specific query formats. Addressing these has prompted the development of approaches that emphasize semantic understanding through vector-based search techniques.

Vector search enables the identification of semantically similar sentences by locating the closest vectors within a high-dimensional space relative to a given query vector. By leveraging this technique, information retrieval systems can achieve greater accuracy and efficiency, retrieving articles that are more contextually relevant to the user’s intent (Monir *et al.* n.d.).

Building on this shift towards semantic understanding, tools like LitSense (Allot *et al.* 2019) and its improved version, LitSense 2.0 (Yeganova *et al.* 2025), represent an early effort to incorporate vector-based techniques into biomedical literature search. These tools begin with traditional term weighting techniques like IDF (Robertson 2004) and BM25 (Robertson *et al.* 1994) to retrieve initial search results, then apply embedding at the sentence and paragraph levels to rerank content based on semantic similarity. While this approach marks progress, the limitation of this method is that it performs semantic comparisons on the traditional term-based results and cannot overcome the inherent challenges of term-based retrieval. Consequently, it fails to retrieve semantically similar articles that were overlooked by the initial term-based approach. Additionally, using data from multiple sources like PubMed and PubMed Central (PMC), LitSense 2.0 seems to retrieve multiple duplicate search results for both sentence and passage searches. These limitations highlight the need for a retrieval framework that integrates semantic understanding more fundamentally, rather than as a post hoc re-ranking step applied to term-based outputs.

Here, we present VectorSage, an advanced biomedical research tool that leverages natural language processing (NLP)-based semantic search in the core search process to overcome the limitations of these search methods and move beyond the constraints of mere keyword-based systems. It offers a searchable database of academic articles’ abstracts from PubMed, allowing users to perform fast and accurate nearest-neighbor searches across this vast dataset. The system employs a hybrid retrieval framework that combines lexical matching with vector-based semantic similarity, allowing it to capture both exact term matches and conceptual relationships between queries and documents. This architecture enables VectorSage to deliver highly relevant search results while efficiently scaling to billions of words in the indexed database. With a user-friendly web interface and downloadable results, it is designed to support a wide range of research workflows. It is publicly accessible online at https://vectorsage.nube.uni-greifswald.de, allowing users to efficiently extract insights from large-scale biomedical literature.

## 2 Methods

### 2.1 Data curation and database creation

To build semantic and keyword indices, PubMed abstracts and metadata were retrieved from the publicly accessible FTP repository (https://ftp.ncbi.nlm.nih.gov/pubmed/) in XML format. The dataset was systematically parsed to extract relevant bibliographic information, resulting in a comprehensive corpus of 38 137 696 entries accessed on January 11, 2025. The data is then stored in a column-oriented relational database. We used DuckDB (DuckDB—an in-process SQL OLAP database management system n.d.) as the database management system, which is an in-process SQL Online Analytical Processing (OLAP) database optimized for high-performance analytical queries.

### 2.2 Embedding and indexing

**Figure 1:**
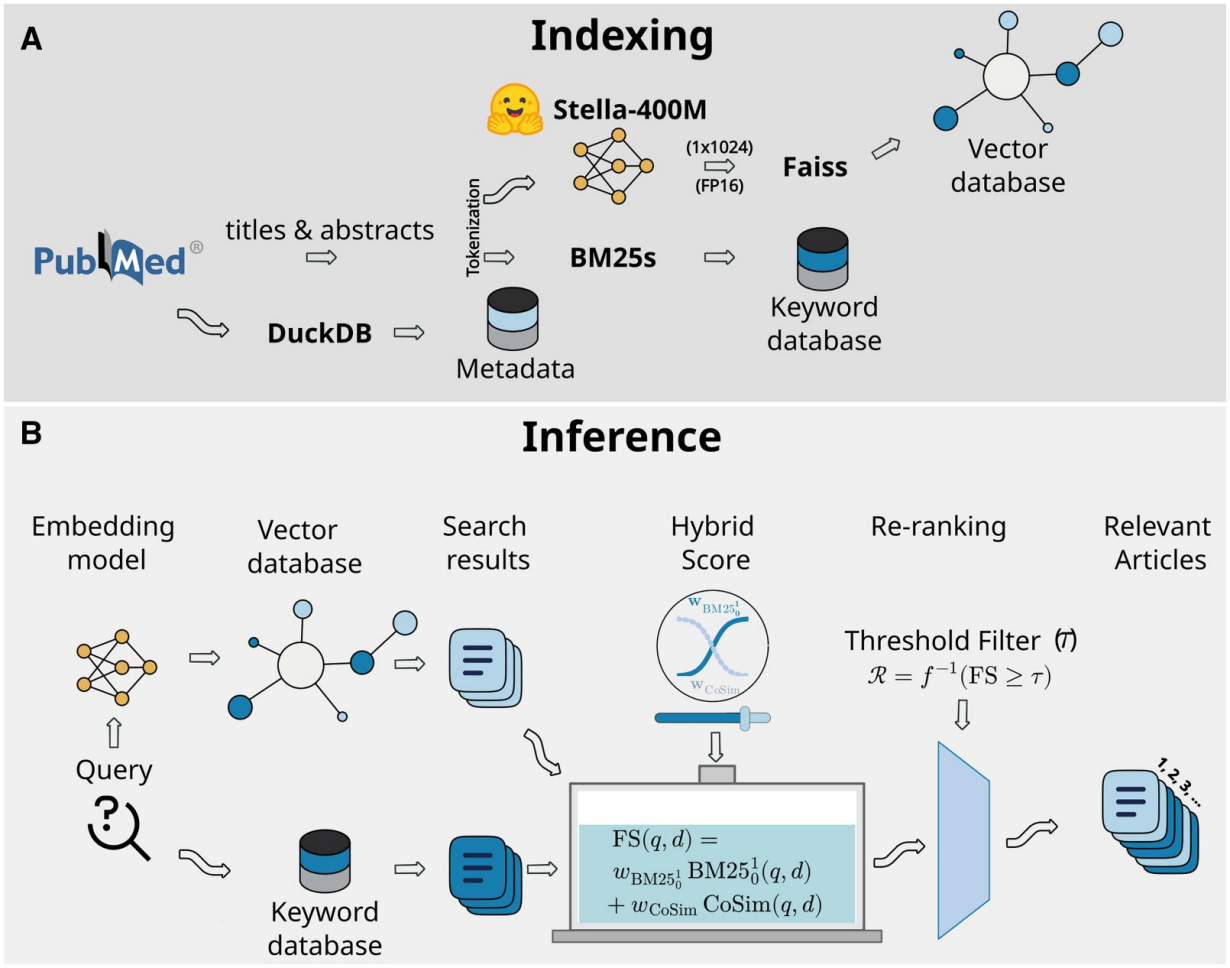
Overview of VectorSage methods. (A) Indexing workflow: The system ingests and filters PubMed data to construct three parallel databases. Titles and abstracts are encoded using the Stella-400M embedding model for storage in a Faiss vector database and simultaneously processed by BM25S for the keyword database. Full records and metadata are structured within DuckDB. (B) Inference workflow: User queries are processed in parallel through the embedding model and keyword database to retrieve initial search results. These results are aggregated using a Final Score (FS) which linearly combines the ${\text{BM}25}_{0}^{1}$ score and Cosine Similarity (CoSim) using adjustable weights ($W_{{\text{BM}25}_{0}^{1}}$ and $W_{\;\text{CoS}\;im}$). Finally, a re-ranking step applies a threshold filter (τ) to return the most relevant articles.

As shown in Fig. 1A, a pre-trained sentence transformer model, Stella-400M (Zhang *et al.* 2024), was selected from the Hugging Face model library to facilitate efficient semantic search. Stella-400M is optimized to enhance similarity between sentence pairs from the same paragraph, based on the assumption that such sentences share closely related semantics using matryoshka representation learning. According to previous benchmarks, the model offers the best balance of performance and computational efficiency, thanks to its relatively low parameter count compared to other large-scale models. Using Stella-400M, numerical vector representations (embeddings) were generated for the abstracts and title, capturing their semantic meaning and contextual nuances.

The Facebook AI Similarity Search (FAISS) library was employed to construct a vector database to support scalable and high-speed retrieval, enabling efficient storage and querying of high-dimensional embeddings (Douze *et al.* 2024). Specifically, we used the FAISS scalar quantizer (SQfp16), which uses a 16-bit floating-point representation of the vector, enabling semantic retrieval through Cosine similarity (CoSim) measures the angular distance between the vector representations of the query q and document d. Cosine similarity originally ranges between −1 and 1; however, in VectorSage a cutoff threshold (t) is applied, ensuring that retained similarity scores are non-negative and fall within the interval [t, 1], and defined as:


$$\text{CoSim}(q,d)=\frac{q\cdot d}{\left\|q\|\|d\right\|}=\frac{\sum_{i=1}^{n}q_{i}d_{i}}{\sqrt{\sum_{i=1}^{n}q_{i}^{2}}\cdot \sqrt{\sum_{i=1}^{n}d_{i}^{2}}}$$

BM25 (Robertson *et al.* 1994), a probabilistic bag-of-words retrieval function, was also integrated to enhance keyword-based search. This implementation allows documents to be ranked based on the occurrence and importance of query terms, regardless of their positional proximity within the text. The BM25 score for a given query q and document d is calculated as:


$$\text{BM}25(q,d)=\sum_{i=1}^{n}\mathrm{IDF}(q_{i})*\frac{f(q_{i},d)*(k_{1}+1)}{f(q_{i},d)+k_{1}*(1-b+b*\frac{\left|d\right|}{\text{avgdl}})}$$

where $f(q_{i},d)$ is the term frequency of term $q_{i}$ in the document d, |d| is the document length and avgdl is the average document length in the corpus. The inverse document frequency (IDF) is given by:


$$\text{IDF}(q_{i})=\log\left(\frac{N-n(q_{i})+0.5}{n(q_{i})+0.5}+1\right)$$

A more efficient variant, BM25S (Lù 2024), was utilized to optimize the performance of our search engine. We used 1024-dimensional vectors for FAISS, and the BM25S vector sizes depended on the vocabulary extracted from each year, as we built separate BM25S for each year’s indices for parallel search. Unlike traditional relational databases, which store structured data in rows and columns, these specialized vector databases are different and excel in capturing nuanced relationships between data points. By organizing data in a multi-dimensional space, these systems enable efficient similarity searches, allowing rapid retrieval of relevant content. To ensure that BM25S scores lie within the [0,1] range, we introduced a modified variant called ${BM25}_{0}^{1}$. This variant applies min–max normalization to the original BM25S scores, scaling them to the interval [0,1] and calculated as:


$${\text{BM}25}_{0}^{1}(d,q)=\frac{\text{BM}25S(d,q)-\underset{d^{\prime }\in D}{\min}\text{BM}25S(d^{\prime },q)}{\underset{d^{\prime }\in D}{\max}\text{BM}25S(d^{\prime },q)-\underset{d^{\prime }\in D}{\min}\text{BM}25S(d^{\prime },q)+\varepsilon }$$

where ε is a very small positive constant introduced to prevent division by zero. This ensures that if all BM25S scores are equal (e.g. when the denominator becomes zero), the normalization does not result in a NaN value.

The final scores (FS) of an article utilize a hybrid approach, combining normalized exact lexical matching and semantic similarity to improve retrieval accuracy and robustness across diverse query types. This ranking is computed as:


$$\text{FS}(q,\;d)=\alpha *{\text{BM}25}_{0}^{1}(q,\;d)+(1-\alpha )*\text{CoSim}(q,\;d)$$

where the weighting parameter α∈[0,1] is determined using a sigmoid-based adaptive function to dynamically balance the contribution of semantic and lexical similarity based on query characteristics:


$$\alpha (d,q)=\frac{1}{1+\exp\left(-a({\text{BM}25}_{0}^{1}(d,q)-b)\right)}$$

In this formulation, a controls the sharpness of the transition, and b defines the midpoint of the sigmoid curve. These parameters were set to a=10 and b=0.5, respectively, to ensure a balanced contribution from both similarity measures across a wide range of query types. The detailed procedure of selecting parameters is provided in Supplementary Material 1 (Appendix A).

### 2.3 Inference

Given a user query q, the tokenizer produces a sequence of tokens $\left\{t_{1},\ldots ,t_{n}\right\}$ with n=|q|. These tokens are used to compute the normalized lexical score ${\text{BM}25}_{0}^{1}(d,q)$ and the semantic similarity score CoSim(q,d) for each document d∈D, as previously defined. Since both scores are scaled to the interval [0,1], they are directly comparable prior to fusion. The adaptive weighting function α∈(q,d) modulates their relative contributions. Thus, the final set of retrieved articles consists of documents whose final score exceeds a predefined threshold τ. Formally, the retrieved set is defined as:


$$R_{\tau }(q)=\{d\in D|\text{FS}(q,d)>\tau \}$$

Because lexical and semantic signals are merged into a single bounded scalar score prior to selection, each document receives exactly one final score, ensuring a unified and conflict-free retrieval outcome following full-paper reranking. In case where two documents receive identical final scores, ties are resolved by prioritizing the document with the higher lexical score, thereby preserving precision for exact term matches. The graphical representation of the inference steps are depicted in Fig. 1B.

A custom Python package was developed to automate the entire indexing pipeline, encompassing embedding generation, vector storage, and database population. This modular and reproducible workflow ensures a scalable and high-performance semantic search system, enabling researchers to efficiently explore large-scale academic datasets with improved precision and contextual relevance. Furthermore, the modular approach facilitates weekly updates to the index, ensuring that the system remains synchronized with PubMed’s latest database releases, thereby maintaining the most up-to-date and comprehensive search capabilities.

### 2.4 VectorSage implementation

VectorSage’s core backend was implemented using PyTorch and Python 3.12 using CUDA 11.8. The VectorSage platform comprises multiple microservices, each responsible for specific computational tasks such as data ingestion, processing, and querying. FastAPI, a modern Python web framework, was used for its ability to deliver high performance with asynchronous support and automatic OpenAPI documentation generation. FastAPI’s native support for asynchronous programming allows VectorSage to handle concurrent tasks, especially the vector similarity search and keyword-based search in parallel, making it particularly well-suited for large-scale systems and efficient for handling large requests.

The front-end of VectorSage is developed using React, a widely adopted JavaScript framework known for its component-based architecture and responsiveness. This modular structure enhances code reusability and maintainability, making the platform adaptable to evolving user needs. For styling the User Interface, Tailwind CSS, a utility-first framework, is employed to create a highly customizable, responsive design. This ensures seamless adaptability across various browsers, providing an optimized, user-friendly experience for users.

## 3 Results

### 3.1 Evaluation and ablation studies

For primary evaluation, we used BioASQ dataset (Krithara *et al.* 2023), a large-scale manually curated Biomedical Question Answering dataset where queries are natural language questions, each associated with human-expert-annotated relevant PubMed articles. BioASQ requires retrieval from large-scale open corpora, closely reflecting real-world search conditions, and frequently exhibits vocabulary mismatch between queries and relevant documents, making it challenging for systems based only on lexical best matching algorithms.

Each query was submitted to LitSense 2.0, PubMed, VectorSage, BM25, and Semantic; and then retrieval performance was assessed using normalized discounted cumulative gain (NDCG), mean average precision (MAP), and mean reciprocal rank (MRR) at rank cutoffs of 5, 10, 15, and 100. As shown in Table 1, VectorSage achieved the highest scores across all metrics on the BioASQ dataset, with differences that are statistically significant. BM25 and the Semantic performed worst, demonstrating that neither lexical nor single-modality dense retrieval is sufficient. Because BioASQ requires systems to retrieve relevant publications directly from large-scale corpora, and it provides a complementary evaluation setting that more closely reflects real-world search scenarios.

**Table 1:** Performance comparison of systems on the BioASQ test dataset.

System	NDCG				MAP				MRR			
	5	10	15	100	5	10	15	100	5	10	15	100
**VectorSage**	0.3632	0.3318	0.3127	0.2883	0.2791	0.2257	0.1986	0.1615	0.5198	0.5284	0.5327	0.5345
**LitSense 2.0**	0.139	0.1326	0.1374	0.2048	0.0863	0.0686	0.0643	0.0738	0.2294	0.2447	0.2503	0.2574
**PubMed**	0.1423	0.1285	0.1244	0.1099	0.0984	0.0786	0.0712	0.0605	0.2391	0.2444	0.2469	0.2474
**BM25**	0.0482	0.0373	0.0307	0.0225	0.028	0.0164	0.0121	0.0077	0.1141	0.1197	0.1201	0.1214
**Semantic**	0.0549	0.0398	0.0339	0.0244	0.0325	0.0182	0.0135	0.0086	0.1312	0.1346	0.1367	0.1379

To further validate the contributions of components, we performed ablation experiments on RELISH (Brown *et al.* 2019), see Supplementary Material 1 (Appendix B) for more detail. We compared semantic-only, lexical-only (BM25) and VectorSage using MAP, MRR and NDCG, and observed similar trend that removing either component consistently degraded performance across all metrics (Supplementary Table S1) compared to combined approach. These results demonstrate that VectorSage’s hybrid framework yields consistently more effective document retrieval and ranking than either purely semantic or sparse lexical retrieval alone.

### 3.2 Real-word case studies

VectorSage is designed for real-world biomedical information retrieval and was evaluated without dataset-specific training or fine-tuning. To assess performance across diverse search scenarios, we constructed a benchmark dataset referred to as UCARE (Use-Case Categorized Application Retrieval Evaluation). The dataset is designed to evaluate retrieval under challenging query conditions frequently encountered in biomedical and public health research. UCARE comprises multiple query categories, each representing a distinct type of challenge. A representative query were created for each category, and categories were systematically designed to vary along dimensions such as semantic specificity, conceptual abstraction, compositional complexity, and domain constraints, thereby reflecting realistic information needs in biomedical literature search. Relevance is approximated using semantic similarity between queries and retrieved content. A detailed description of the dataset, including category definitions and the full list of queries, is provided in Supplementary Material 1 (Appendix C and D) and the repository (see Data Availability).

For evaluation, LitSense 2.0 was assessed using its passage-mode search. Cosine similarity was calculated between each query and the top K retrieved passages (K = 3), as this mode returns passages from across the full text rather than being limited to abstracts. Cosine similarity is used here explicitly as a proxy measure of relevance rather than a standard information retrieval metric. For PubMed and VectorSage, cosine similarity was computed between each query and the abstracts of the top K retrieved documents. Figure 3 presents the comparative evaluation of VectorSage, LitSense 2.0, and PubMed under this experimental setting.

As shown in Fig. 3A, VectorSage consistently achieves higher mean cosine similarity between queries, and their top K retrieved results across nearly all UCARE query categories. Figure 3B further shows that, when averaged across all categories, the overall mean cosine similarity reaches 0.629 for VectorSage, compared with 0.151 for LitSense 2.0 and 0.123 for PubMed, indicating substantially semantic alignment between user queries and retrieved literature. At the category level, VectorSage maintains similarity score above the 0.5 relevance threshold in most cases, demonstrating robust retrieval performance across queries characterized by terminology gaps, conceptual abstraction, and multi-concept reasoning. See Supplementary Material 1 (Appendix C and D) for detail about the categories and analysis. In contrast, both LitSense 2.0 and PubMed show weak retrieval performance with variability across query categories, with several query categories returning no results. A similar trend is observed in the relevance distribution of retrieved documents. As shown in Fig. 3C, VectorSage retrieves the highest proportion of Relevant (45%) and Partially Relevant (52%) documents, with only 3% classified as irrelevant. By comparison, LitSense 2.0 and PubMed frequently failed to return any results, accounting for 73% and 79% of cases, respectively.

Together, these results demonstrate that the hybrid retrieval strategy implemented in VectorSage improves robustness across diverse biomedical query types, particularly in scenarios where relevant literature may be conceptually related but lexically dissimilar to the user query.

### 3.3 Web tool and features

**Figure 2:**
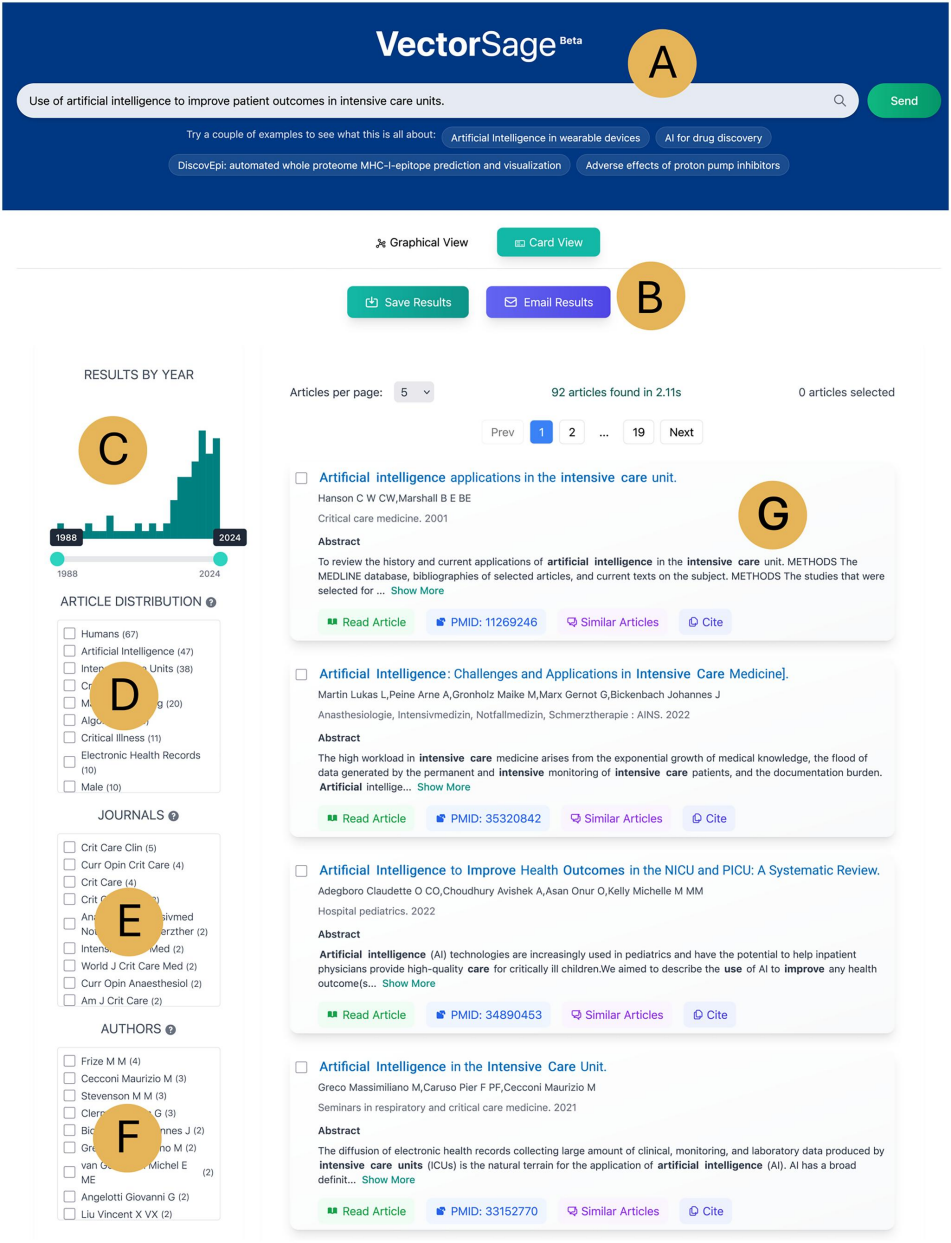
User interface of VectorSage. (A) Users can enter search queries in the top search bar or explore pre-loaded example queries. (B) Search results can be exported in various file formats for downstream analysis. To refine the results, multiple filtering options are provided, including: (C) year of publication, (D) associated MeSH terms, (E) journal name, and (F) author name. (G) Each article is displayed as an interactive card containing detailed metadata such as PubMed ID, title, abstract, DOI, and citation options. The card also includes a feature to find similar articles, and clicking on the card reveals additional details.

VectorSage provides an intuitive web interface (see Fig. 2) for searching academic literature that is relevant to their research interests. The platform is accessible through any modern web browser at https://vectorsage.nube.uni-greifswald.de/ and offers a streamlined search experience. At present, the system retrieves articles from disciplines indexed in PubMed. In forthcoming updates, additional publicly available databases, including prominent preprint repositories, will be indexed and integrated into the platform.

**Figure 3:**
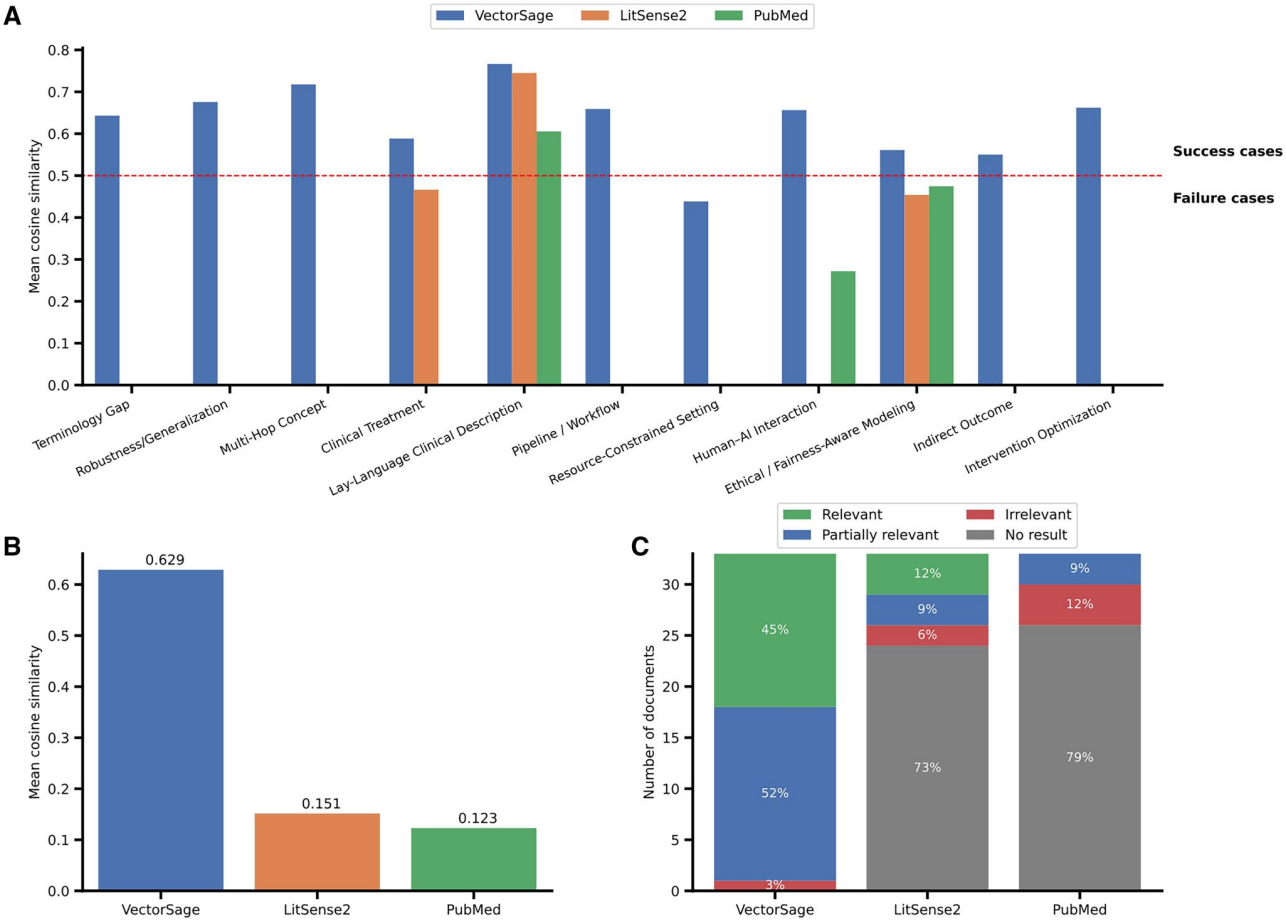
Comparative evaluation of VectorSage, LitSense 2.0, and PubMed on the UCARE dataset. (A) Mean cosine similarity between each query and the top three retrieved results across systems. The x-axis shows the UCARE query categories, and the y-axis indicates the mean cosine similarity of the top three retrieved results. Empty bars indicate cases where a system returned no results for the given query. (B) Overall mean cosine similarity aggregated across all query categories for each system. (C) Distribution of document-level relevance outcomes across systems, categorized as Relevant, Partially Relevant, Irrelevant, and No Results. Stacked bars represent the percentage of retrieved documents in each category.

#### 3.3.1 Search functionality

Users can input queries in the form of keywords, phrases, or full sentences describing their research interests or the title of an article. These queries are converted into vector representations using a sentence-transformer model, enabling semantic comparison with pre-embedded academic abstracts.

The search currently operates on the titles and abstracts of peer-reviewed academic articles. Upon searching, the search results are displayed in a ranked or sorted list as a card view based on their FS, ensuring that users receive the most relevant literature recommendations. Each search result includes essential metadata such as the title, abstract, author names, journal source, and unique identifiers (PMID, DOI), as illustrated in Fig. 2G.

#### 3.3.2 Similar articles functionality

Beyond the core searches, VectorSage also provides an iterative exploration mechanism through a “Similar Articles” search feature, see Fig. 2G. Users can refine their search and discover closely related publications by selecting this option, which retrieves additional articles with high semantic similarity to the chosen reference abstract. This function facilitates exploratory literature review, particularly beneficial for interdisciplinary research where exact keyword matches may be insufficient.

#### 3.3.3 Filtration of results

To further enhance the precision and efficiency of information retrieval in line with the user’s needs, VectorSage implements a robust filtering system that enables users to refine their search results based on multiple parameters. One key filtering mechanism allows users to constrain the publication timeline, selecting articles within a defined temporal range as shown in Fig. 2C. This feature ensures that users can focus on the most relevant and recent research developments within their field.

Additionally, VectorSage incorporates a categorization framework that organizes articles based on the Top 10 MeSH (Medical Subject Headings) terms, Journals, and Authors’ article count found in the retrieved results, see Fig. 2C–F. This structured classification facilitates domain-specific exploration, allowing researchers to navigate literature aligned with their areas of interest. Users can also customize the number of displayed records per session, optimizing accessibility and workflow efficiency.

#### 3.3.4 Export options

VectorSage provides users with flexible options for downloading the data they have queried or browsed. There are two main ways to export results based on the user’s needs. First, each article card features a “Cite” button, allowing users to copy or download the article citation quickly, as shown in Fig. 2G. Second, for bulk downloads, users can select multiple articles and click the “SAVE” button above the data table on the search results or display page, as shown in Fig. 2B. This provides three options:

- Download the selected articles.
- Download all articles on the current view page.
- Download the complete set of articles.

Any of these options can be used to download data in various formats, including CSV and BibTeX. These export options support the structured integration of search results into citation management systems and facilitate downstream analysis for large-scale data exploration.

## 4 Discussion

In this study, we introduced VectorSage, an advanced biomedical search framework that integrates semantic search capabilities with traditional keyword-based retrieval to improve the accuracy, flexibility, and accessibility of academic literature discovery. Unlike conventional platforms such as PubMed, which primarily rely on exact keyword matching, VectorSage is designed to operate across both lexical and semantic dimensions. It achieves this through a dual-objective hybrid architecture that simultaneously ranks articles based on keyword relevance and semantic similarity, embedding both queries and articles in a shared vector space to better align with user intent.

While PubMed continues to serve as a foundational resource for biomedical literature, its dependency on MeSH terms presents inherent limitations. Additionally, inconsistencies in MeSH term assignment can reduce the discoverability of newly published articles. These challenges are compounded by a broader shift in user behavior, increasingly influenced by the widespread adoption of large language models (LLMs), AI assistants, AI agents, and tools such as ChatGPT. These systems promote natural language interaction, lowering barriers to information access, and changing expectations around how scientific content should be searched. In contrast, effective use of PubMed often requires users to construct structured queries using Boolean operators, field tags, and filters to retrieve more specific or comprehensive results. This query syntax, while powerful, introduces a steep learning curve and stands in contrast to the more conversational and flexible querying experience enabled by modern language technologies.

PubMed’s current search architecture, including its ATM system, MeSH, and Best Match ranking algorithm, was designed to enhance retrieval within a keyword-driven paradigm. However, these mechanisms have not fully kept pace with evolving user behavior that favors concept-based, conversational queries. This growing mismatch highlights the need for search systems that are more adaptable to language variability and systems that can better interpret how users naturally express their queries.

Recent advances in LLMs, including domain-adapted transformer encoders such as BioBERT, PubMedBERT, SapBERT, E5-Large, and SPECTER2, have demonstrated strong performance in semantic representation and biomedical retrieval tasks (Lee *et al.* 2020, Gu *et al.* 2021, Singh *et al.* 2023, Khattab *et al.* 2026, Liu *et al.* n.d.). These models highlight the potential of embedding-based methods to capture conceptual relationships beyond exact keyword overlap, enabling improved retrieval for queries expressed in natural or descriptive language. However, relying solely on semantic embeddings is insufficient for many biomedical and clinical queries, where precise terminology, such as gene symbols, drug names, clinical abbreviations, or rare disease terms, plays a critical role in identifying relevant literature (Schulz and Juric 2020, Ma *et al.* n.d.). In such contexts, purely semantic retrieval may overlook documents containing the exact terminology required by the query. Further, deploying such models for large-scale indexing and real-time retrieval may require substantial computational resources, which can limit their applicability in routine clinical or academic environments.

In response to these evolving search behaviors and the limitations of traditional systems, we developed VectorSage to bridge the gap between user expectations and existing biomedical search infrastructure. By leveraging semantically enriched representations with traditional lexical retrieval methods, VectorSage facilitates retrieval even when there is minimal lexical overlap between user queries and relevant literature. Furthermore, VectorSage is designed around a compact encoder within a hybrid lexical–semantic retrieval framework, enabling efficient large-scale indexing, minimal hardware requirements, and zero-shot operation without task-specific fine-tuning while maintaining competitive retrieval performance. Importantly, the VectorSage architecture is model-agnostic and can incorporate alternative embedding models, including larger biomedical encoders or future LLM-based representations, when sufficient computational resources are available.

Users interact with biomedical search systems in diverse ways; some submit concise, title-like queries that depend on exact keyword matches, while others use natural language to express broader, more conceptual information needs. To support this spectrum of query formulations, VectorSage integrates a global ranking mechanism that balances lexical relevance with semantic similarity. This approach addresses the trade-off between the precision of keyword-based methods and the generalization strength of semantic embeddings. By mitigating conflicting signals between the two, the system more effectively interprets user intent and consistently ranks the most relevant articles at the top of the results list. This was further supported by the evaluation results, where VectorSage successfully retrieved articles that were conceptually related yet lexically dissimilar to the query. In contrast, keyword-based systems showed reduced performance for natural language–style queries that lack explicit biomedical keywords, resulting in no returned results for several queries. LitSense 2.0 applies semantic reranking after an initial lexical retrieval stage; consequently, if relevant documents are not retrieved during this initial candidate generation step, the semantic reranker cannot subsequently recover them. Similarly, PubMed relies primarily on keyword matching augmented by ATM to controlled vocabularies such as MeSH, which may be less effective when queries use indirect phrasing, combine multiple concepts, or describe higher-level processes that do not map directly to a single indexed term. These findings highlight the advantage of hybrid retrieval for real-world search scenarios, enabling VectorSage to interpret diverse query formulations, generalize across heterogeneous query types, and consistently retrieve relevant articles.

Nevertheless, the current version of VectorSage also inherits some limitations. Like most embedding-based models, it can struggle with queries involving explicit logical negation (for example, “no,” “not”) or complex Boolean composition. It may retrieve documents that match the core context of the query while insufficiently respecting exclusion conditions, consistent with prior work showing that embeddings often underrepresent negation and other logical operators (Petcu *et al.* 2025, Weller *et al.* n.d.). Finally, the fusion mechanism of VectorSage uses fixed, analytically chosen weighting parameters rather than dataset specific empirically tuned; although this yields predictable, model and data-gnostic behavior for general biomedical queries, it may not be optimal for niche domains (for example, rare diseases, gene variants etc.). However, we can leverage the accumulated user relevance feedback to guide domain specific adaptation in subsequent releases of VectorSage.

## 5 Conclusion

VectorSage is publicly available as a web-based tool at https://vectorsage.nube.uni-greifswald.de/, requiring no local installation or programming expertise. Its user-friendly interface supports researchers across domains in accessing and navigating biomedical literature more effectively. As the community increasingly demands tools that support natural language input and semantic exploration, VectorSage represents a practical and forward-compatible advancement in biomedical information retrieval.

Looking ahead, we recognize that the exponential growth of biomedical publications imposes increasing demands on search systems, making the optimization of search algorithms for speed, relevance ranking, and scalability increasingly critical. Future improvements in VectorSage will focus on refining re-ranking mechanisms and leveraging newer and advanced embedding models for similarity search to enhance semantic understanding. Additionally, we are exploring more efficient indexing strategies, such as hierarchical navigable small-world (HNSW) graphs and vector quantization, to improve retrieval speed and computational efficiency. Further, future iterations of VectorSage aim to expand the search index to full-text articles, incorporate additional metadata fields, and integrate with external repositories such as arXiv and bioRxiv. Users will then have the flexibility to search exclusively within specific peer-reviewed sources, such as PubMed, or to broaden the scope to include preprint literature, ensuring comprehensive coverage from early-stage findings to fully validated research.

The VectorSage team welcomes feedback and suggestions at any time. For inquiries, feature requests, or comments, please get in touch with us via the feedback form at https://vectorsage.nube.uni-greifswald.de/.

## Supplementary Material

vbag116_Supplementary_Data

## Data Availability

The data underlying this article are derived from sources on the public domain. PubMed abstracts and metadata are publicly available through NCBI FTP repository at https://ftp.ncbi.nlm.nih.gov/pubmed. VectorSage is freely available to all users without login at https://vectorsage.nube.uni-greifswald.de. The code and evaluation data underlying this article are available at https://github.com/Mehdilotfi7/VectorSage_Supp.
