# Supplementary material for: VectorSage: enhancing PubMed article retrieval with advanced semantic search
Source: Bioinform Adv. 2026 Apr 24;6(1):vbag116. doi: 10.1093/bioadv/vbag116 (PMC13188980; doi:10.1093/bioadv/vbag116)
Supplement: vbag116_Supplementary_Data [file vbag116_supplementary_data.docx]

**Supplementary Material for VectorSage: Enhancing PubMed Article Retrieval with Advanced Semantic Search**

Yasas Wijesekara^1,†^, Rahul Brahma^1,†^, Mehdi Lotfi^1^, Marcus Vollmer^1^, Lars Kaderali^1,*^

1. Institute of Bioinformatics, University Medicine Greifswald, Felix-Hausdorff-Str. 8, 17475 Greifswald, Germany

† Yasas Wijesekara and Rahul Brahma contributed equally to this work.

- To whom correspondence should be addressed. Email: [lars.kaderali@uni-greifswald.de](mailto:lars.kaderali@uni-greifswald.de)

**Appendix A: Mathematical Justification of the Adaptive Sigmoid Weighting**

**Problem Formulation**

Our retrieval framework combines normalized lexical matching and semantic similarity through an adaptive weighting mechanism. For a query [
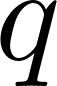
](https://www.codecogs.com/eqnedit.php?latex=q#0) and document [
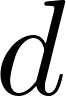
](https://www.codecogs.com/eqnedit.php?latex=d#0), define the normalized lexical score as

[
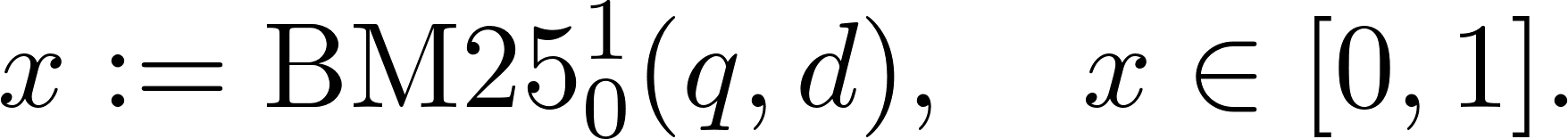
](https://www.codecogs.com/eqnedit.php?latex=x%20%3A%3D%20%5Cmathrm%7BBM25%7D_%7B0%7D%5E%7B1%7D(q%2Cd)%2C%20%5Cquad%20x%20%5Cin%20%5B0%2C1%5D.#0)

and Cosine similarity (CoSim) of the
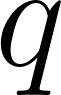
 and
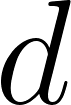
 is defined as:


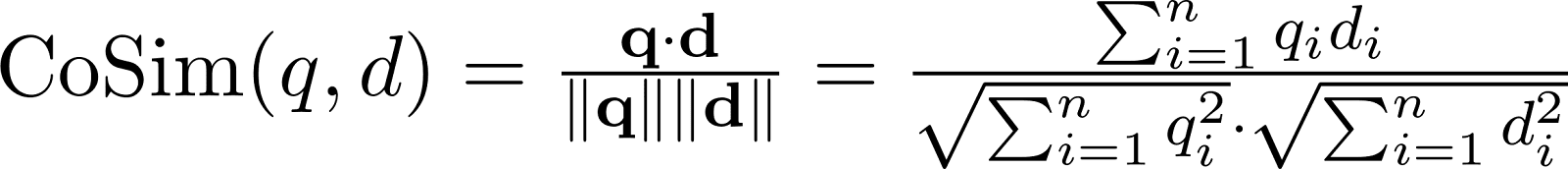


The final score (FS) used in the main method is

[
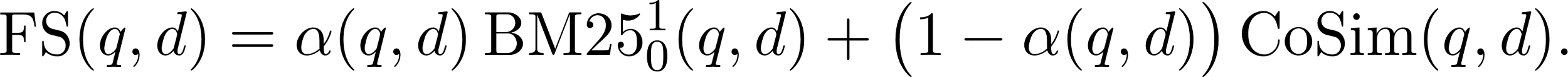
](https://www.codecogs.com/eqnedit.php?latex=%5Cmathrm%7BFS%7D(q%2Cd)%3D%5Calpha(q%2Cd)%5C%2C%5Cmathrm%7BBM25%7D_%7B0%7D%5E%7B1%7D(q%2Cd)%2B%5Cbig(1-%5Calpha(q%2Cd)%5Cbig)%5C%2C%5Cmathrm%7BCoSim%7D(q%2Cd).#0)

The adaptive lexical weight depends on the normalized BM25 value through

[
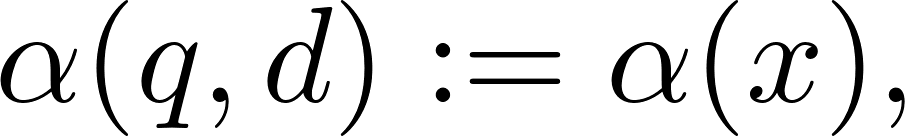
](https://www.codecogs.com/eqnedit.php?latex=%5Calpha(q%2Cd)%3A%3D%5Calpha(x)%2C#0)

where

[
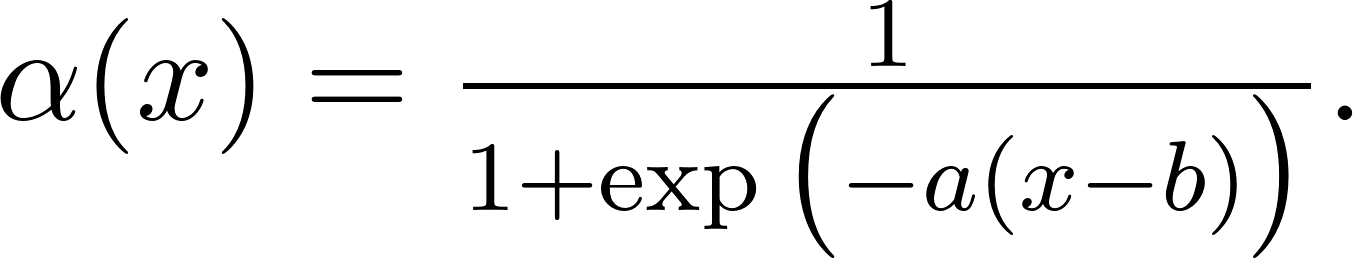
](https://www.codecogs.com/eqnedit.php?latex=%5Calpha(x)%3D%5Cfrac%7B1%7D%7B1%2B%5Cexp%5Cbig(-a(x-b)%5Cbig)%7D.#0)

The parameters [
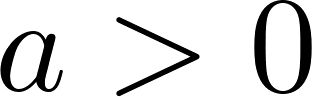
](https://www.codecogs.com/eqnedit.php?latex=a%3E0#0) and [
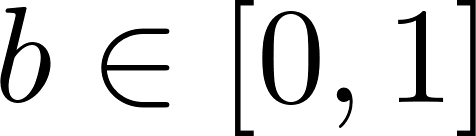
](https://www.codecogs.com/eqnedit.php?latex=b%5Cin%5B0%2C1%5D#0) control the steepness and midpoint of the transition. We now justify the choice

[
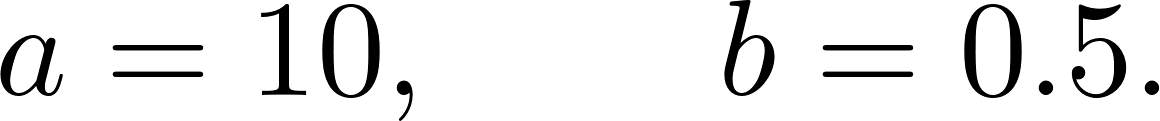
](https://www.codecogs.com/eqnedit.php?latex=a%3D10%2C%20%5Cqquad%20b%3D0.5.#0)

**Basic Properties of the Weight Function**

**Boundedness**

*Proposition.* For all [
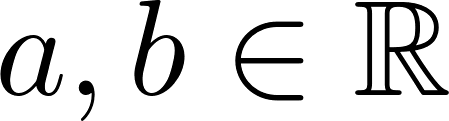
](https://www.codecogs.com/eqnedit.php?latex=a%2Cb%5Cin%5Cmathbb%7BR%7D#0) and all [
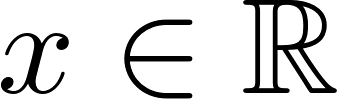
](https://www.codecogs.com/eqnedit.php?latex=%20x%5Cin%5Cmathbb%7BR%7D#0),

we have

[
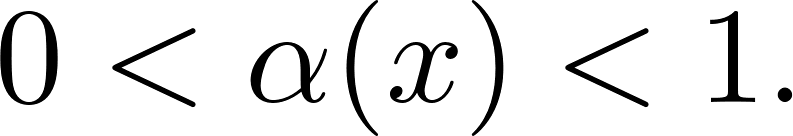
](https://www.codecogs.com/eqnedit.php?latex=0%3C%5Calpha(x)%3C1.#0)

*Proof*. Since

[
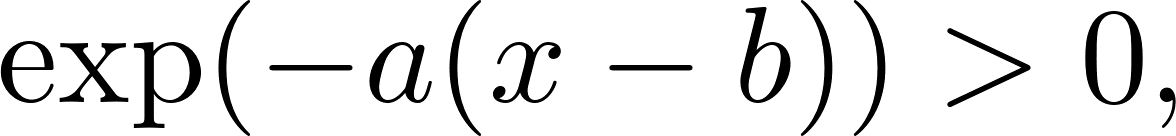
](https://www.codecogs.com/eqnedit.php?latex=%5Cexp(-a(x-b))%3E0%2C#0)

it follows that

[
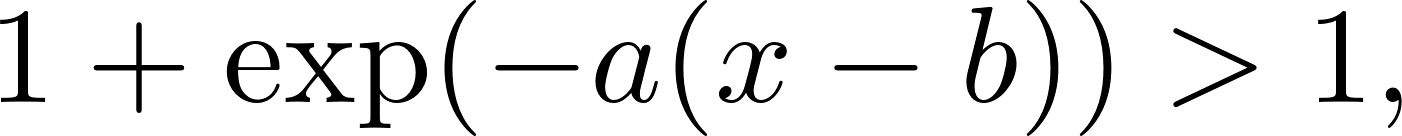
](https://www.codecogs.com/eqnedit.php?latex=1%2B%5Cexp(-a(x-b))%3E1%2C#0)

and therefore

[
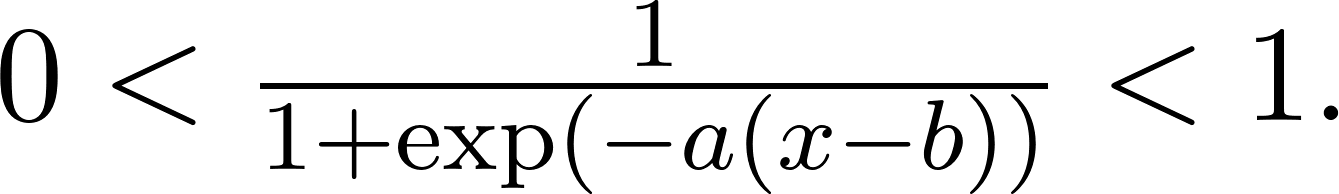
](https://www.codecogs.com/eqnedit.php?latex=0%3C%5Cfrac%7B1%7D%7B1%2B%5Cexp(-a(x-b))%7D%3C1.#0)

Thus,
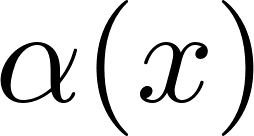
 always defines a valid convex weight and guarantees that the final score remains a bounded convex combination of lexical and semantic similarities.

**Uniqueness of the Midpoint Parameter**

*Proposition.* The parameter
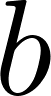
 determines the balance point at which lexical and semantic contributions are equal.

*Proof*. Evaluating at [
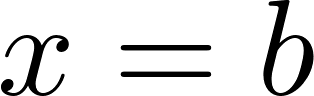
](https://www.codecogs.com/eqnedit.php?latex=x%3Db#0) gives

[
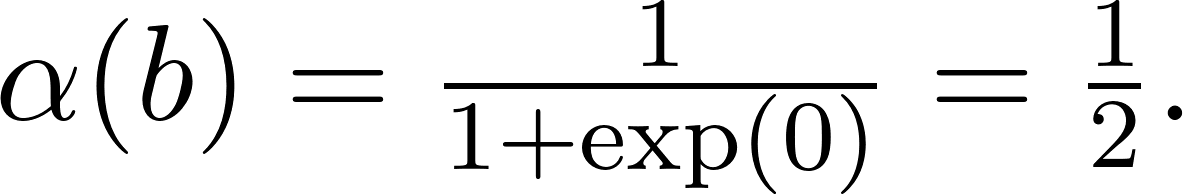
](https://www.codecogs.com/eqnedit.php?latex=%5Calpha(b)%3D%5Cfrac%7B1%7D%7B1%2B%5Cexp(0)%7D%3D%5Cfrac%7B1%7D%7B2%7D.#0)

Thus when

[
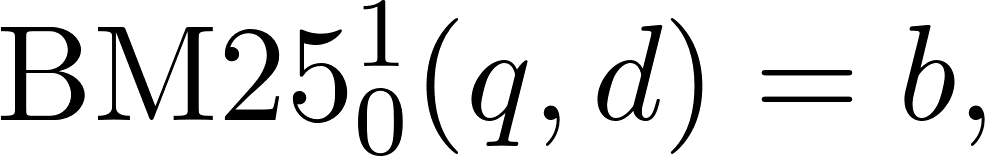
](https://www.codecogs.com/eqnedit.php?latex=%5Cmathrm%7BBM25%7D_%7B0%7D%5E%7B1%7D(q%2Cd)%3Db%2C#0)

lexical and semantic contributions are equal.

**Symmetry Argument**

Because [
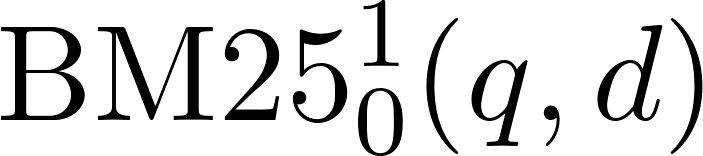
](https://www.codecogs.com/eqnedit.php?latex=%5Cmathrm%7BBM25%7D_%7B0%7D%5E%7B1%7D(q%2Cd)#0) is normalized to the interval [
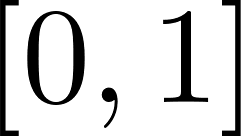
](https://www.codecogs.com/eqnedit.php?latex=%5B0%2C1%5D#0), and no prior bias toward lexical or semantic dominance is imposed, the fusion mechanism should be symmetric with respect to this interval.

The unique symmetric point is

[
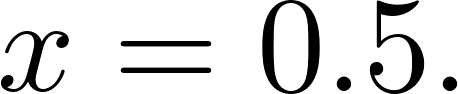
](https://www.codecogs.com/eqnedit.php?latex=x%3D0.5.#0)

Therefore, the balance point must satisfy

[
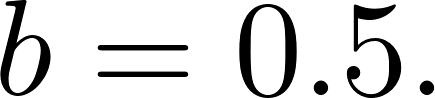
](https://www.codecogs.com/eqnedit.php?latex=b%3D0.5.#0)

Any other value would introduce an explicit preference toward either lexical or semantic weighting.

**Endpoints Tolerance Analysis**

Since the sigmoid cannot reach exact values [
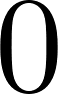
](https://www.codecogs.com/eqnedit.php?latex=0#0) or [
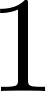
](https://www.codecogs.com/eqnedit.php?latex=1#0) for finite inputs, we impose approximate constraints:

[
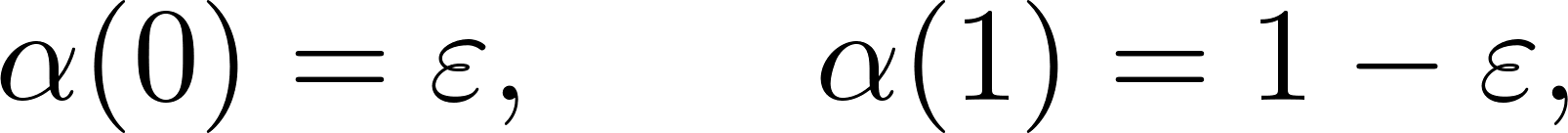
](https://www.codecogs.com/eqnedit.php?latex=%5Calpha(0)%3D%5Cvarepsilon%2C%20%5Cqquad%20%5Calpha(1)%3D1-%5Cvarepsilon%2C#0)

where

[
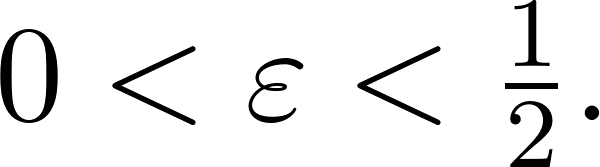
](https://www.codecogs.com/eqnedit.php?latex=0%3C%5Cvarepsilon%3C%5Cfrac%7B1%7D%7B2%7D.#0)

Using the logit transformation,

[
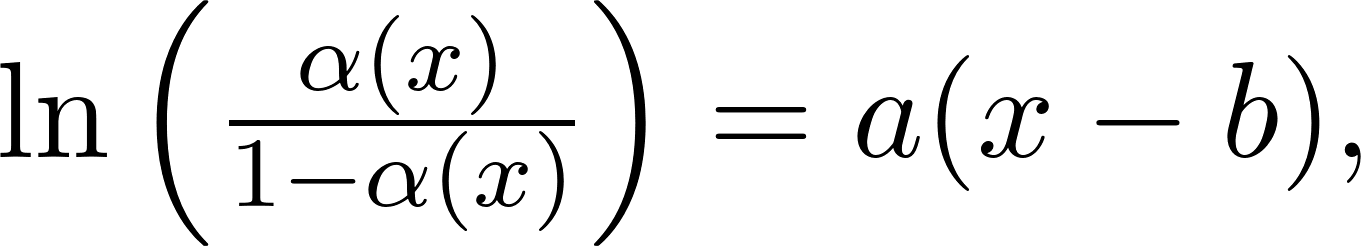
](https://www.codecogs.com/eqnedit.php?latex=%5Cln%5Cleft(%5Cfrac%7B%5Calpha(x)%7D%7B1-%5Calpha(x)%7D%5Cright)%3Da(x-b)%2C#0)

and substituting the endpoint conditions yields

[
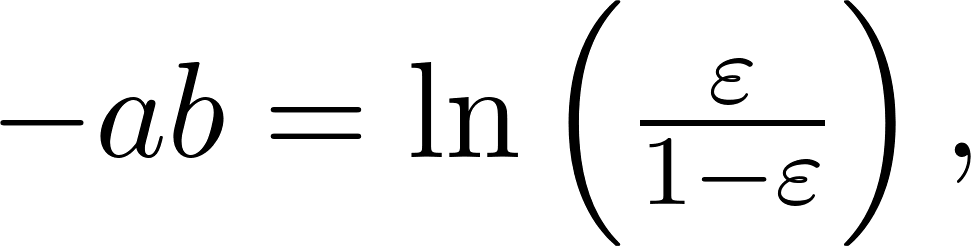
](https://www.codecogs.com/eqnedit.php?latex=-ab%3D%5Cln%5Cleft(%5Cfrac%7B%5Cvarepsilon%7D%7B1-%5Cvarepsilon%7D%5Cright)%2C#0) [
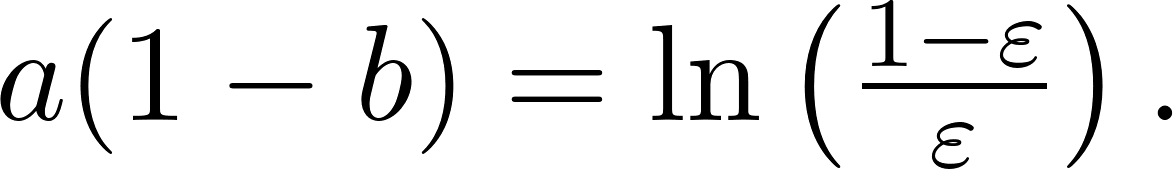
](https://www.codecogs.com/eqnedit.php?latex=a(1-b)%3D%5Cln%5Cleft(%5Cfrac%7B1-%5Cvarepsilon%7D%7B%5Cvarepsilon%7D%5Cright).#0)

Adding both equations gives

[
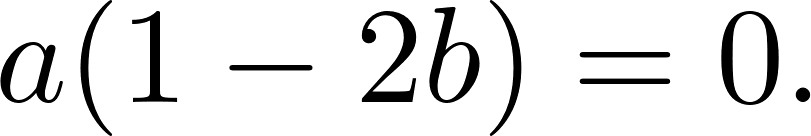
](https://www.codecogs.com/eqnedit.php?latex=a(1-2b)%3D0.#0)

Since [
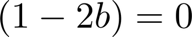
](https://www.codecogs.com/eqnedit.php?latex=(1-2b)%3D0#0) so [
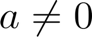
](https://www.codecogs.com/eqnedit.php?latex=a%5Cnot%3D0#0). Substituting [
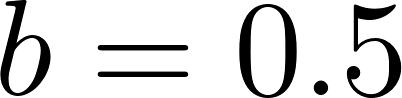
](https://www.codecogs.com/eqnedit.php?latex=b%3D0.5#0) in [
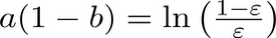
](https://www.codecogs.com/eqnedit.php?latex=a(1-b)%3D%5Cln%5Cleft(%5Cfrac%7B1-%5Cvarepsilon%7D%7B%5Cvarepsilon%7D%5Cright)#0) yields

[
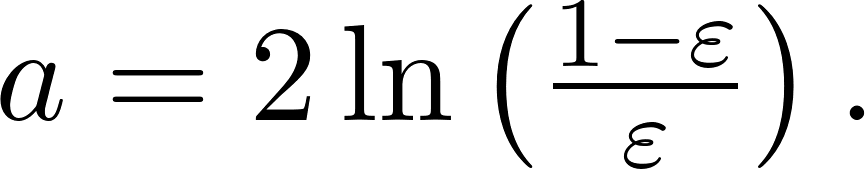
](https://www.codecogs.com/eqnedit.php?latex=a%3D2%5Cln%5Cleft(%5Cfrac%7B1-%5Cvarepsilon%7D%7B%5Cvarepsilon%7D%5Cright).#0)

Thus, once the tolerance [
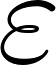
](https://www.codecogs.com/eqnedit.php?latex=%5Cvarepsilon#0) is chosen, the steepness parameter [
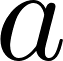
](https://www.codecogs.com/eqnedit.php?latex=a#0) is uniquely determined.

**Choice of** [**
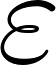
**](https://www.codecogs.com/eqnedit.php?latex=%5Cvarepsilon#0) **and Resulting Parameters**

We select a small tolerance such that the lexical weight is near zero for minimal lexical evidence and near one for strong lexical matches, while maintaining a smooth transition. Choosing

[
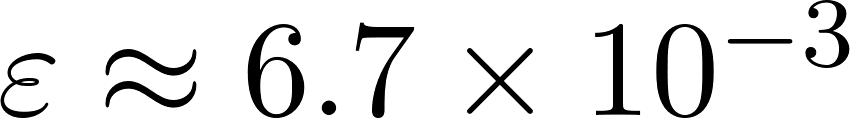
](https://www.codecogs.com/eqnedit.php?latex=%5Cvarepsilon%20%5Capprox%206.7%5Ctimes10%5E%7B-3%7D#0)

gives

[
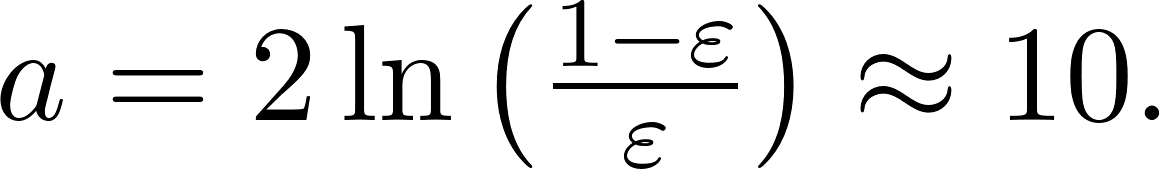
](https://www.codecogs.com/eqnedit.php?latex=a%3D2%5Cln%5Cleft(%5Cfrac%7B1-%5Cvarepsilon%7D%7B%5Cvarepsilon%7D%5Cright)%5Capprox10.#0)

Under this configuration,

[
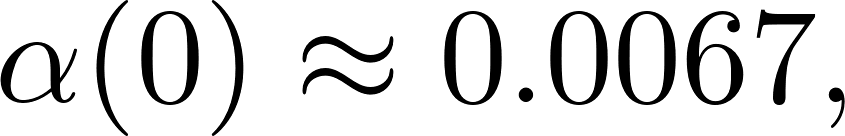
](https://www.codecogs.com/eqnedit.php?latex=%5Calpha(0)%5Capprox0.0067%2C#0) [
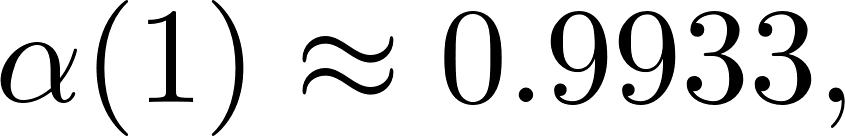
](https://www.codecogs.com/eqnedit.php?latex=%5Calpha(1)%5Capprox0.9933%2C#0) [
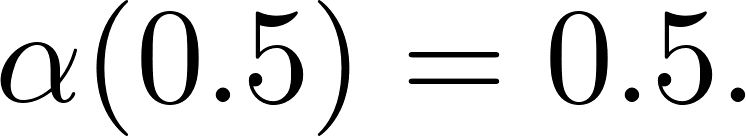
](https://www.codecogs.com/eqnedit.php?latex=%5Calpha(0.5)%3D0.5.#0)

This ensures a smooth yet decisive transition between semantic and lexical dominance while preserving numerical stability.

**Final Form Used in the Main Method**

The adaptive weighting function used in the retrieval model is

[
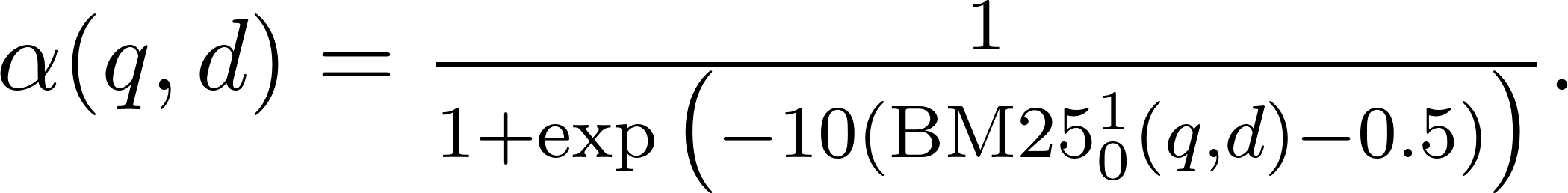
](https://www.codecogs.com/eqnedit.php?latex=%5Calpha(q%2Cd)%3D%5Cfrac%7B1%7D%7B1%2B%5Cexp%5Cbig(-10(%5Cmathrm%7BBM25%7D_%7B0%7D%5E%7B1%7D(q%2Cd)-0.5)%5Cbig)%7D.#0)

The parameter [
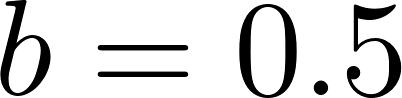
](https://www.codecogs.com/eqnedit.php?latex=b%3D0.5#0) follows from symmetry and balance considerations, while [
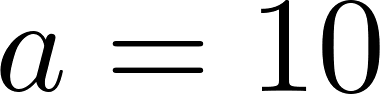
](https://www.codecogs.com/eqnedit.php?latex=a%3D10#0) corresponds to the tolerance level above and yields effective and stable lexical–semantic fusion.

As both parameters are established through formal mathematical derivation rather than empirical tuning, they are independent of the document collection or query domain and thus generalizable across diverse information retrieval corpora.

**Appendix B: Ablation details**

To evaluate the individual contributions of each retrieval component within the hybrid retrieval system, we conducted an ablation study using the RELISH dataset. In RELISH, each seed article serves as a query within an article-to-article retrieval framework, where candidate documents were automatically generated using established lexical retrieval approaches named as Article-based PubMed Search Engine (APSE) which is built on three baseline methods namely PubMed Related Articles, BM25, and TF–IDF, and subsequently merged into a fixed pool of 60 candidate articles per query prior to human-expert annotating each candidate as relevant, partial relevant, or irrelevant. Since the candidate pool is derived exclusively from lexical-based methods, systems employing lexical-based retrieval inherently benefit from this construction, as their retrieved documents are more likely to overlap with the pre-annotated pool, potentially inflating their performance relative to semantic retrieval components.

Retrieval effectiveness was assessed using Mean Average Precision (MAP), Mean Reciprocal Rank (MRR) and Normalized Discounted Cumulative Gain (NDCG) at cutoffs of 5, 10 and 100.

To assess the contribution of each retrieval component, we performed an ablation analysis of three VectorSage configurations on the subset split reported in [1]. In the lexical-only configuration, semantic retrieval was disabled and ranking was based exclusively on BM25. In the semantic-only configuration, BM25 was disabled and retrieval was performed using FAISS-based approximate nearest-neighbour search over Stella400m_v5 embeddings. In the hybrid configuration, VectorSage combined results from both components using a sigmoid-based score-merging function, thereby integrating complementary signals from sparse lexical matching and dense semantic similarity.

As shown in Table S1, VectorSage consistently outperformed both BM25 and Semantic system on all metrics and at all evaluated cutoffs. For example, NDCG@5 improved from 0.7734 with BM25-only to 0.8604, while MAP@5 increased from 0.8384 to 0.9074, reflecting substantially enhanced precision among the documents. Similar trends were observed for MRR, with VectorSage achieving 0.9653 at cutoff of 5 compared to 0.9279 for BM25 and 0.9625 for the semantic‑only configuration, indicating that the first relevant document is found earlier on average when both components are active. These ablation results demonstrate that lexical and semantic modules make independent, complementary contributions, and that their integration within VectorSage yields better performance than either purely semantic or purely lexical retrieval system alone. This integration improves robustness and supports effective retrieval across diverse retrieval scenarios.

Table S1: Ablation analysis evaluating the individual and combined contributions of VectorSage retrieval components on RELISH dataset.

| **System** | **NDCG** | | | **MAP** | | | **MRR** | | |
| --- | --- | --- | --- | --- | --- | --- | --- | --- | --- |
|  | 5 | 10 | 100 | 5 | 10 | 100 | 5 | 10 | 100 |
| VectorSage | 0.8604 | 0.8245 | 0.9057 | 0.9074 | 0.8573 | 0.8223 | 0.9653 | 0.9661 | 0.9667 |
| Semantic | 0.8461 | 0.8196 | 0.8979 | 0.8964 | 0.8516 | 0.8107 | 0.9625 | 0.9625 | 0.9637 |
| BM25 | 0.7734 | 0.7450 | 0.8668 | 0.8384 | 0.7821 | 0.7702 | 0.9279 | 0.9313 | 0.9320 |

Metrics are computed per query and mean over participating queries

**Appendix C. UCARE dataset, systematic category design, and success/failure cases**

Rather than assembling a set of conventional keyword-oriented questions, UCARE was organized around predefined query categories, each representing a distinct retrieval challenge. This design makes it possible to compare systems under controlled semantic conditions and to determine whether retrieval differences arise from genuine differences in system capability rather than from inconsistent query construction. The full query list is provided in repository [2], and Table S2 summarizes the category definitions used in the present evaluation.

Table S2. Category definitions used in UCARE.

| **Category** | **Definition** |
| --- | --- |
| Terminology Gap | Queries that express a known concept using synonyms, alternate phrasing, or uncommon terminology relative to how it appears in papers. |
| Multi-Hop Concept Queries | Queries that require linking two or more concepts, where relevance depends on their combination. |
| Clinical Treatment Queries | Queries centered on treatment decisions, therapy selection, treatment response, comparative effectiveness, dosing or management strategies, or clinical pathways, often phrased in a problem-oriented way rather than drug or procedure names. |
| Lay Language Clinical Description | Queries describing clinical conditions, symptoms, or care processes using non-technical or everyday language. |
| Pipeline / Workflow Queries | Queries describing an end-to-end process rather than one isolated method. |
| Resource-Constrained Setting Queries | Queries that emphasize limited data, limited infrastructure, sparse surveillance, or low-resource clinical or public health environments. |
| Robustness / Generalization Queries | Queries focused on performance stability across hospitals, countries, scanners, time periods, coding practices, or reporting regimes. |
| Human–AI Interaction Queries | Queries about how humans use AI outputs, including interpretability, uncertainty communication, workflow integration, clinician override, trust, and usability. |
| Ethical / Fairness-Aware Modeling Queries | Queries addressing bias, unequal error rates, representativeness, subgroup performance, fairness constraints, or distributional harms. |
| Indirect Outcome Queries | Queries targeting proxy or surrogate signals used to infer a latent clinical or epidemiological phenomenon. |
| Intervention Optimization Queries | Queries that ask for selecting or optimizing interventions under constraints, often implying tradeoffs and objective functions. |

**Query design rationale**

The individual UCARE queries were written so that each category stresses a different aspect of retrieval. For example, the Terminology Gap query asks “*What makes some patients with the same tumor type respond well to checkpoint blockade while others do not benefit at all?*”, which intentionally avoids more standard article-level terminology such as immune checkpoint inhibitor response, resistance, predictive biomarkers, or tumor microenvironment. Likewise, in the Clinical Treatment category, the query “*What strategies reduce the risk of kidney damage in patients who need to receive large amounts of contrast dye for imaging?*” frames the problem of reducing kidney damage in patients receiving contrast dye, rather than directly naming canonical terms such as contrast-induced nephropathy or contrast-associated acute kidney injury. These formulations were chosen to test whether a retrieval system can recover semantically relevant literature even when the query does not contain the dominant indexing or title words likely to appear in published papers.

Other categories were designed to stress retrieval in different ways. The Robustness/Generalization query asks whether a sepsis detection algorithm trained before a major event can remain accurate after changes in patient mix and care pathways, which targets a literature often phrased with terms such as temporal drift, distribution shift, transportability, and external validation rather than the everyday wording used in the query. The Human–AI Interaction Queries category similarly uses decision-oriented wording such as when clinicians trust or override an alert, while the relevant literature often uses terms like clinical decision support, physician override, workflow integration, trust calibration, or alarm fatigue. These query designs are especially challenging for systems that depend on exact or near-exact lexical overlap. All remaining categories follow the same rationale outlined in the table and are designed to stress-test a retrieval system under realistic real-world conditions.

## **Appendix D. Success and failure definition**

As shown in Figure 3(A), retrieval performance for each category was interpreted using a threshold on mean cosine similarity. Categories with a mean cosine similarity above 0.5 were classified as success cases, whereas those below 0.5 were classified as failure cases. This threshold-based interpretation provides a simple category-level view of whether a system was able to retrieve semantically aligned results for a given query formulation.

**Category-level interpretation of success and failure**

Figure 3 (A) shows that VectorSage achieved success in nearly all UCARE categories. Mean cosine similarity remained above the 0.5 threshold for Terminology Gap, Robustness/Generalization, Multi-Hop Concept, Clinical Treatment , Lay-Language Clinical Description, Pipeline/Workflow , Human–AI Interaction, Ethical/Fairness-Aware Modeling, Indirect Outcome, and Intervention Optimization Queries. The only category that fell below the threshold was Resource-Constrained Setting Queries. This pattern suggests that VectorSage can maintain semantic retrieval quality across categories in which the query wording is broader, more descriptive, more compositional, or more weakly aligned with standard biomedical indexing terms.

The performance of VectorSage in the Terminology Gap and Robustness/Generalization categories is particularly informative, as both rely heavily on natural-language query formulations that differ from the specialized phrasing typically used in the literature. In these settings, the query expresses the underlying concept in intuitive or descriptive language rather than in the technical terminology most often found in articles. For example, a question asking why patients with the same tumor type respond differently to checkpoint blockade does not point to a single fixed term, but to a broader clinical concept that may be approached in the literature from multiple directions, including biomarker heterogeneity, tumor immune microenvironment, resistance mechanisms, patient stratification, or response prediction. Similarly, a query about whether a sepsis detection model remains accurate after a major event may correspond to literature framed in terms such as temporal drift, distribution shift, transportability, or external validation. Keyword-first systems are vulnerable in such cases because they depend heavily on lexical overlap between the query and the indexed text, whereas hybrid retrieval can recover documents that are conceptually aligned even when the wording differs substantially. This likely explains why VectorSage performs well in these categories, whereas the keyword-first systems failed to retrieve any result at all as shown in Figure 3 (A).

VectorSage which is a hybrid retrieval system performs strongly in categories such as Multi-Hop Concept, Pipeline/Workflow, and Intervention Optimization Queries, which are not difficult because of synonymy alone, but because the query expresses a compound information need. For example, the gut microbiota and immunotherapy query requires linking microbial composition, treatment context, and response prediction, while the ICU admission query requires reasoning about triage, resource scarcity, and critically ill patients.

Keyword-based systems often retrieve documents that match only one fragment of such queries, failing to capture the full intent of the information needed. By contrast, hybrid retrieval is better positioned to preserve the combined semantic meaning of the entire query and therefore retrieve results that align more closely with the question as a whole.

By contrast, LitSense 2.0 shows a much narrower success profile in Figure 3 (A). It exceeds the 0.5 threshold only in Lay-Language Clinical Description, while the remaining evaluated categories fall below the threshold. This result is consistent with its retrieval design, in which lexical retrieval is used to generate candidates before semantic reranking is applied. If semantically relevant documents are not retrieved in the initial keyword-oriented stage, the semantic reranker cannot recover them later. As a result, categories characterized by abstraction shifts, compositional queries, or low lexical overlap are more likely to fail.

The relatively better performance of LitSense 2.0 in Lay-Language Clinical Description likely reflects the fact that some descriptive clinical expressions still preserve enough overlap with standard disease or symptom terminology to survive the initial lexical retrieval stage. However, this partial success does not generalize to categories such as Robustness/Generalization, Human–AI Interaction, or Ethical/Fairness-Aware Modeling, where relevant papers often use specialized technical vocabulary drawn from machine learning, clinical informatics, or health systems research rather than the broader wording used in the query.

PubMed shows the weakest category-level profile overall, with success only in Lay-Language Clinical Description and failure across all other categories shown in Figure 3A. This behavior is consistent with its dependence on keyword matching augmented by Automatic Term Mapping to MeSH and related translation tables. PubMed can often improve recall when a query maps cleanly to a known MeSH term or entry term, but this mechanism is still fundamentally limited when the query uses indirect phrasing, combines several concepts, or asks about process-level or systems-level issues that do not map neatly onto a single controlled vocabulary term.

The Robustness/Generalization category illustrates this limitation well. The query about a sepsis detection algorithm remaining accurate after a pandemic does not use standard retrieval anchors such as distribution shift, temporal drift, external validation, or transportability, even though those are the terms likely to appear in the literature. PubMed and LitSense 2.0 are therefore disadvantaged because the query omit the canonical lexical forms that would guide initial retrieval, whereas VectorSage can use semantic proximity to bridge from the descriptive wording of the query to the technical language of the relevant papers.

The lower performance of VectorSage on Resource-Constrained Setting Queries reflects the fact that this category was designed to test a particularly demanding form of retrieval, in which relevance depends not only on the clinical problem but also on strict operational constraints. The query asks how hospitals with limited laboratory capacity can accurately identify patients with a life-threatening infection without advanced testing equipment. This was intentionally formulated to move beyond standard sepsis or infection-diagnosis retrieval and instead probe whether a system can preserve the practical context that makes evidence usable in low-resource settings.

This makes the retrieval problem unusually difficult. Accurate identification of life-threatening infection is typically supported by laboratory measurements, microbiological testing, blood-based biomarkers, or other infrastructure-dependent diagnostics. By explicitly restricting advanced testing, the query narrows the relevant literature toward approaches based on minimal-input or non-invasive data, such as physiological monitoring, signal-based models, or other resource-feasible screening strategies. At the same time, the query still asks for accurate identification, which raises the bar further because many such alternatives remain experimental, incompletely validated, or not yet routinely deployable in hospitals.

The retrieved results suggest that VectorSage captures the broad semantic domain of severe infection diagnosis, but has more difficulty prioritizing documents that also satisfy the tighter constraints of low-resource applicability and non-laboratory feasibility. In this sense, the category serves as a useful stress test: it distinguishes systems that retrieve generally related clinical literature from systems that can preserve the full operational meaning of a real-world query.

Overall, the category-level analysis supports that the systems fail for different structural reasons. PubMed is most limited by its dependence on explicit biomedical terminology and term mapping. LitSense 2.0 improves this by introducing semantic reranking but still depends on a lexical-first candidate generation step, which restricts recall in semantically challenging categories. VectorSage is more robust because it integrates lexical and semantic evidence in parallel, rather than in a stepwise manner as in LitSense 2.0. Using the Final Score function described in the main paper, it combines both signals through an adaptive sigmoidal weighting scheme. This design allows VectorSage to better handle queries that rely heavily on natural language phrasing, as well as queries that express compound information needs spanning multiple concepts

**References**

[1] Ravinder R, Geist L, Rebholz-Schuhmann D, Castro LJ. Datasets for OntoClue Project. [accessed 2026 Feb 28]. https://zenodo.org/records/14801641. doi:10.5281/ZENODO.14801641

[2] Mehdilotfi7/VectorSage_Supp. [accessed 2026 Mar 6]. https://github.com/Mehdilotfi7/VectorSage_Supp
